# L'otospongiose: étude rétrospective à propos de 36 cas

**DOI:** 10.11604/pamj.2014.18.242.3509

**Published:** 2014-07-23

**Authors:** Brahim Bouaity, Mehdi Chihani, Mohammed Touati, Youssef Darouassi, Karim Nadour, Haddou Ammar

**Affiliations:** 1 Service d'Oto-Rhino-Laryngologie et de Chirurgie Cervico-Faciale, Hôpital Militaire Avicenne, Marrakech, Maroc

**Keywords:** Otospongiose, surdité, platinectomie, platinotomie, otospongiosis, deafness, stapedectomy, stapedotomy

## Abstract

L'objectif de l’étude est de rapporter notre expérience concernant la prise en charge de l'otospongiose et comparer nos résultats avec ceux de la littérature. Nous rapportons une étude rétrospective concernant 36 cas d'otospongiose colligés au service d'Oto-rhino-laryngologie de l'hôpital militaire Avicenne de Marrakech, entre Janvier 2009 et Décembre 2012. L’âge moyen des patients était de 38 ans avec une nette prédominance masculine (77%). Notre étude a révélé un retard diagnostique (stades audiométriques II et III). Ce diagnostic est le plus souvent simple, très fortement suspecté dès la première consultation devant l'existence d'une surdité de transmission ou mixte à tympan normal qui constitue le signe de découverte le plus prépondérant et constant dans notre série (100%), accompagnée parfois d'acouphènes (41%). L'existence d'antécédents familiaux de la maladie a été retenue chez 33,33%. L'audiométrie tonale liminaire et la tympanométrie ont été réalisées chez tous nos patients (100%) évoquant le diagnostic dans la majorité des cas. La tomodensitométrie, non obligatoire, reste indispensable pour le diagnostic différentiel et surtout permet de prévoir les difficultés opératoires ainsi que les éventuelles associations pathologiques. Tous les patients ont subit une intervention chirurgicale comportant soit une platinectomie partielle ou totale(83%) ou une platinotomie. L’évolution et les résultats ont été généralement satisfaisants, par ailleurs nous avons constaté la survenue d'un cas de fistule périlymphatique, la persistance des acouphènes chez six patients et une surdité de transmission chez trois patients.

## Introduction

L′otospongiose est une ostéodystrophie de la capsule otique [1]. C'est une maladie génétique de transmission autosomique dominante à pénétrance variable et à prédominance féminine. Elle survient chez 0,2 à 1% de la population [2]. Cliniquement, le diagnostic est suspecté devant une surdité de transmission ou mixte acquise ou évolutive à tympan normal [3]. L′audiométrie tonale liminaire, et particulièrement l′impédancemétrie, est fondamentale mettant en évidence l'absence du réflexe stapédien ou d′un effet on/off. Même si le diagnostic est essentiellement clinique, la tomodensitométrie reste utile pour éliminer les diagnostics différentiels et déceler les variations anatomiques ainsi que les éventuelles associations pathologiques. Le traitement est chirurgical et la technique adoptée est différente selon les auteurs (platinotomie, platinectomie) [4]. Parfois, le traitement peut faire appel à la prothèse auditive. Le but de ce travail est de rapporter notre expérience en matière de prise en charge de cette pathologie et de comparer nos résultats avec ceux de la littérature.

## Méthodes

Notre étude est rétrospective, portant sur 36 cas d'otospongiose, colligés dans le serviced'oto-rhino-laryngologie de l'hôpital militaire Avicenne de Marrakech, sur une période de 4 ans, s’étendant du 1er Janvier 2008 au 31 Décembre 2011. Les patients avec une cophose, les patients dont le suivi post opératoire n'a pas été effectué de manière satisfaisante et consensuelle, notamment les patients avec des données clinico-radiologiques indisponibles ainsi que les patients n'ayant pas bénéficié d'une cure chirurgicale ou perdus de vue, ont été exclus de l’étude. Pour cela, une fiche d'exploitation a été établie, visant à préciser les profils épidémiologiques, cliniques, thérapeutiques et évolutifs des malades.

## Résultats

L’âge de nos patients variait entre 25 et 60 ans, avec une moyenne d’âge de 38 ans. Nous avons noté une prédominance masculine (77% des cas). Il relève de notre étude un retard diagnostique (Stades audiométriques II et III). La surdité constitue le signe de découverte le plus fréquent dans notre série, présent dans 100% des cas (Tableau 1). Un cas similaire dans la famille a été retrouvé chez 12 patients. Tous nos patients ont bénéficié d′une audiométrie tonale liminaire ainsi que d'une impédancemétrie dont le but est de confirmer qu'il s'agit d'une surdité de transmission ou mixte ainsi que de déterminer le stade évolutif selon le degré de la perte auditive moyenne (PAM) (Tableau 2). Le réflexe stapédien était absent dans 84% des cas, alors que dans 16% des cas l'effet on-off était présent.

**Tableau 1:** Circonstances de découverte dans notre série

Signes d'appel	pourcentage
Hypoacousie	100%
Acouphène	41%
Vertige	17%
Paraacousie de Willis	2%

**Tableau 2:** Perte auditive moyenne

Perte auditive moyenne	Pourcentage (%)
20 < PAM <39	6%
40 < PAM < 69	61%
70 < PAM < 89	27%
90 < PAM < 119	6%
PAM > 119	0%

Dans notre étude la tomodensitométrie a été demandée chez vingt six malades. Elle était normale chez vingt deux patients. Elle a objectivé une petite hypodensité préstapedienne bilatérale chez deux malades et une hypodensité péricochléaire et de la fenêtre ovale dans deux autres cas. Tous les patients ont bénéficié d'une intervention chirurgicale. La voie d'abord endaurale à minima ou de Shambaugh a été retenue pour tous les patients. La platinectomie a été réalisée chez 30 malades, elle était partielle dans 28% des cas, et totale dans 55% des cas. Quatre malades ont bénéficié d'une platinotomie avec mise en place d'un greffon, la platinotomie calibrée n'a été effectuée que chez deux cas. Le choix du système transmetteur a été porté pour les pistons pour tous les opérés. Quelques incidents opératoires sont survenus au cours des interventions: une déchirure tympanique chez deux patients, une déchirure du lambeau tympanoméatal chez deux autres et une luxation de l'enclume dans un cas. L’évolution a été marquée par la survenue d'un cas de dysgueusie transitoire et de fistule périlymphatique, la persistance des acouphènes chez six patients, et surdité de transmission chez trois patients. Elle a été favorable dans les cas restants.

## Discussion

**Figure 1:**
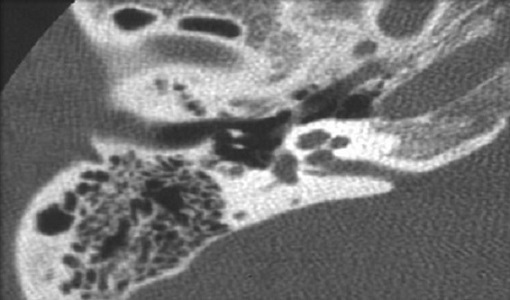
TDM en coupe axiale montrant une hypodensité préstapedienne

**Figure 2:**
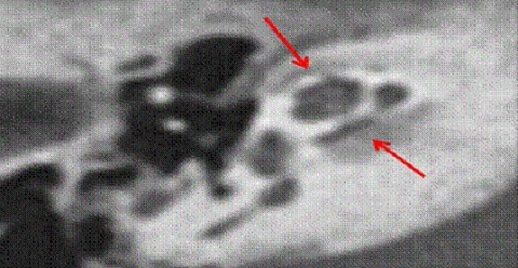
TDM en coupe axiale montrant une hypodensité péricochléaire et de la fenêtre ovale

L′otospongiose est l'une des principales étiologies des surdités acquises de l′adulte: des études autopsiques systématiques retrouvent 8 à 12% de foyers otospongieux alors que l′incidence clinique oscille entre 0,2 et 1% [2, 5]. La moyenne d’âge de découverte de l'otospongiose dans notre série était de 38 ans, avec des extrêmes allant de 25 à 60 ans. Selon Ayach D [6], la moyenne d’âge était entre 30 et 40 ans. Pour S.Benarab [7], elle était de 40 ans avec des extrêmes allant de 26 à 60 ans. Nous avons noté une prédominance masculine de 77%, ce qui ne concorde pas avec les données de la littérature. Et cela s'explique par la prédominance masculine dans la population militaire. Pour Ayach D [6], le sexe ratio était de 2. Pour S.Benarab [7], il était de 2,12. La fréquence des antécédents familiaux est variable selon les auteurs, elle varie de 40 à 50% [8]. Certains l′estiment à 30%, d′autres comme Millman à 50%. Dans notre étude 33,33% des malades ont des cas similaires dans la famille, ce qui concorde avec les données de la littérature. La surdité constitue le motif essentiel de consultation [1]. Elle s′accompagne fréquemment d′acouphènes. Dubreuil a retrouvé des acouphènes dans 40% des cas [9]. Dans notre série, la surdité était présente chez tous nos patients, tandis que les acouphènes étaient présents dans 41% des cas (Tableau 3). L'audiométrie tonale liminaire permet de classer l′otospongiose en quatre stades selon la classification de Juers et de Shambaugh. Dans notre série, on a noté que 18% de nos malades présentaient un stade I, 40% un stade II, 41% un stade III et 10% un stade IV. On conclut que la plupart des malades ne sont pris en charge qu′aux stades II et III. La tomodensitométrie (TDM) confirme le diagnostic clinique en montrant les foyers hypodensespérilabyrinthiques de l′os temporal [10, 11]. Elle permet d′éliminer une autre cause de surdité de transmission à tympan normal et de réaliser un bilan anatomique pré chirurgical. L′intérêt de l′imagerie par résonance magnétique (IRM) réside essentiellement dans l′exploration des complications chirurgicales non résolues par le scanner [12]. Dans notre étude, la TDM a été réalisée chez vingt six patients. Elle est normale chez vingt deux malades. Elle a objectivé deux cas d'hypodensité préstapedienne (Figure 1) et deux autres cas d'hypodensité péricochléaire (Figure 2).

**Tableau 3:** Circonstances de découverte rapportées dans la littérature

Signes d'appel	Ayach D [6]	Dubreuil [9]	Notre série
Surdité	75%	85%	100%
Acouphène	30%	40%	41%
Vertige	-	10 à 25%	17%
Paraacousie de Willis	-	10%	2%

Dans notre série, aucun patient n'a bénéficié d'une imagerie par résonance magnétique. Le traitement chirurgical de l′otospongiose constitue l′option de choix dans la prise en charge thérapeutique. Plusieurs techniques peuvent être réalisées: platinectomie partielle ou totale et platinotomie avec interposition ou calibrée. Il n′y a actuellement pas de méta-analyse faisant la synthèse des résultats de la chirurgie de l'otospongiose. Aucune étude de ce type ne permet actuellement d′affirmer qu′une technique opératoire est inférieure ou supérieure à une autre. L'analyse des grandes séries historiques et plus récentes de la littérature montre une stabilité des résultats depuis de nombreuses années [4]. Dans notre série, la platinectomie a été pratiquée trente fois, elle est partielle dans 28% des cas, et totale dans 55% des cas. La platinotomie avec mise en place d'un greffon a été réalisée chez quatre malades, et la platinotomie calibrée a été effectuée chez deux autres. L′appareillage prothétique représente une alternative intéressante à la chirurgie lorsque celle-ci est contre-indiquée ou refusée. Dans notre étude, aucun patient n'a bénéficié d'un appareillage. Le risque de complications post opératoires est à prendre en compte et doit être expliqué au patient, en particulier celui de cophose post opératoire qui est évalué entre 0,5 et 1% des interventions. Les autres complications sont dominées par les acouphènes, une paralysie faciale, une dysgueusie souvent réversible, des vertiges, luxation de l'enclume, fistule périlymphatique et une perforation de la membrane tympanique. Ces complications peuvent être source de conflit avec les patients lorsqu′elles ne sont pas expliquées préalablement à l′intervention, et que les alternatives thérapeutiques n’étaient pas présentées et commentées. Cette compréhension et l′acceptation par le patient des risques opératoires sont des points importants. L′information du patient doit être la plus claire et la plus complète possible en se fondant sur les données de la littérature médicale. Le recueil d′un consentement éclairci est indispensable [4]. Dans notre série quelques incidents sont survenus au cours de l'intervention: une déchirure tympanique chez deux patients, une déchirure du lambeau tympanoméatal chez deux autres et une luxation de l'enclume chez un cas. L’évolution a été marquée par la survenue d'un cas de dysgueusie transitoire et de fistule périlymphatique, la persistance des acouphènes chez six patients, surdité de transmission chez trois patients, et aucun cas de labyrinthisation n'a été déplorée.

## Conclusion

L'otospongiose est une affection fréquente qui se caractérise par une surdité de transmission à tympan normal en absence de pathologie associée. Son diagnostic est facile à retenir, mais les patients ne consultent qu’à un stade audiométrique II et III. Le traitement de l'otospongiose est essentiellement chirurgical, son principe consiste à retirer l’étrier ankylosé avec une platinectomie (partielle ou totale) ou une platinotomie (calibrée ou avec interposition) et à le remplacer par une prothèse, interposée entre l'enclume et la fenêtre ovale afin de restaurer la transmission des sons aux liquides de l'oreille interne. L'appareillage prothétique représente une alternative intéressante à la chirurgie lorsque celle-ci est contre-indiquée ou refusée par le patient.
